# Microstructural Modification of TiAl6V4 Alloy to Avoid Detrimental Effects Due to Selective In Vivo Crevice Corrosion

**DOI:** 10.3390/ma15165733

**Published:** 2022-08-19

**Authors:** Maria Herbster, Karsten Harnisch, Paulina Kriegel, Andreas Heyn, Manja Krüger, Christoph H. Lohmann, Jessica Bertrand, Thorsten Halle

**Affiliations:** 1 Institute of Materials and Joining Technology, Otto-von-Guericke University, 39106 Magdeburg, Germany; 2 Department of Orthopaedic Surgery, Otto-von-Guericke University, 39120 Magdeburg, Germany

**Keywords:** TiAl6V4, heat treatment, α and β phase, selective dissolution, crevice corrosion, inflammatory conditions, in vitro test method, mechanical properties, wear resistance, cell adhesion

## Abstract

TiAl6V4 wrought alloy is a standard material used for endoprostheses due to its ideal characteristics in terms of osseointegration. However, the insufficient wear and crevice corrosion resistance of TiAl6V4 are limiting factors that can cause clinical problems. Therefore, the objective of this study was to analyze and identify suitable phases and microstructural states of TiAl6V4 alloy with advantageous implant properties by thermal treatments. By varying the temperature and cooling rate, four heat treatment strategies were derived that produced different microstructural states that differed in morphology, arrangement and proportions of phases present. All TiAl6V4 modifications were characterized regarding their microstructure, mechanical, corrosive and tribological properties, as well as cell adhesion. The acicular, martensitic microstructure achieves a significant hardness increase by up to 63% and exhibits improved corrosion and wear resistance compared to the forged condition. Whereas the modified microstructures showed similar electrochemical properties in polarization tests using different electrolytes (PBS with H_2_O_2_ and HCl additives), selective α or β phase dissolution occurred under severe inflammatory crevice conditions after four weeks of exposure at 37 °C. The microstructurally selective corrosion processes resemble the damage patterns of retrieved Ti-based implants and provide a better understanding of clinically relevant in vivo crevice corrosion mechanisms. Furthermore, a microstructural effect on cell attachment was determined and is correlated to the size of the vanadium-rich β phase. These key findings highlight the relevance of an adapted processing of TiAl6V4 alloy to increase the longevity of implants.

## 1. Introduction

Ti-based alloys exhibit excellent biocompatibility that promotes osseointegration and possess an exceptional strength to weight ratio and mechanical properties, which favor their use as a metallic implant material. They are of great importance as the main permanently implanted metallic biomaterials for cementless implant components, which represent the current gold standard in hip arthroplasty. In 2020, 78% of all primary hip replacements in Germany had a cementless fixation based on the Arthroplasty Registry [1]. In this scope, TiAl6V4 wrought alloy (ISO 5832-3 [2], ASTM F136 (ELI) [3], ASTM F1472 [4]) is the most commonly used implant alloy. It is conventionally applied in a thermo-mechanically processed state with a recrystallized, fine-grained, two-phase microstructure.

Different explant studies documented non-mechanically caused corrosion damage at the modular taper junction of retrieved TiAl6V4 implant components, resulting in the release of wear particles, corrosion products and metal ions into the periprosthetic tissue, which impairs the service life of endoprostheses and can cause premature revision surgeries [5,6,7,8]. Various damage causes as well as hypotheses have been identified for the observed in vivo degradation of TiAl6V4, including hydrogen embrittlement, pitting and crevice corrosion, as well as inflammatory effects. The passive layer’s integrity may be impaired by factors such as tribological effects (e.g., fretting) or oxidative stress (OS) involving hydrogen peroxide (H_2_O_2_), hydroxyl and hydroperoxyl radicals [9]. OS can occur during inflammation of periprosthetic tissue, which can be temporary (e.g., after implantation) or chronic (e.g., debris-related) [10]. An inflammatory reaction causes an acidic environment in which peroxides are produced that can further accelerate degradation and dissolution of Ti-based alloys [7,11,12].

The specific conditions of these complex in vivo degradation mechanisms are not entirely understood and have not yet been duplicated in the laboratory. For implant material improvements, inflammatory conditions have to be simulated using a combination of acidulated physiological solution with hydrogen peroxide addition [13,14]. Different studies showed that proteins, such as albumin, in combination with H_2_O_2_ significantly harm the corrosion resistance of TiAl6V4 alloy [13,15]. In this context, the present TiAl6V4 microstructure is a relevant influencing factor whose effect must be studied and adapted for its advantageous properties.

Microstructural modifications of TiAl6V4 alloy offer tremendous potential for improvements as microstructural constituents determine the mechanical, tribological, technological and electrochemical properties. Thermal and thermomechanical treatments can generate a wide range of morphology and arrangements of the phases (α, α′ and β) present [16]. Effective processes for property modification are solution annealing, recrystallization, aging, stress relief annealing and thermomechanical treatments. They differ in the adjustment of the annealing temperature, holding time, cooling conditions and degree of pre-deformation [16].

Based on the available literature, there are two basic thermal treatment strategies to modify the microstructure of TiAl6V4 alloy, resulting in increased mechanical properties. On the one hand, microstructure refinement and particle hardening in a process of solution treatment and aging (STA) can achieve strengthening [17,18,19,20]. On the other hand, the size and volume fraction of the reinforcing β phase is intended to increase by β annealing. The presence of the β phase in Ti-based alloys is advantageous for biomedical implant applications because it has a very low Young’s modulus compared to other metallic implant alloys [21,22,23,24]. The available studies on TiAl6V4 alloy for biomedical application primarily focus on the mechanical characterization of different microstructures. Detailed investigations on the microstructural effects on corrosion behavior under physiological conditions are limited and show divergent results [8,25,26,27,28]. Furthermore, the lack of appropriate standards for biomedical corrosion testing prevents a comparison due to differing test conditions (e.g., test fluids, surface treatment). In addition, critical, physiologically relevant conditions, such as inflammatory species or crevice microenvironments, are not currently considered in the implant approval process, and their effects during long-term implantation are poorly understood. A systemic examination of different TiAl6V4 heat treatments on corrosion and wear resistance as a function of microstructure has not been carried out. In this scope, we hypothesize that the implant properties of TiAl6V4 can be significantly improved by an adapted microstructure. In this study, we derive suitable thermal treatment strategies for TiAl6V4 and assess different microstructure–property relations relevant for implant application. The following research questions are intended to be answered:

- What influence do the microstructural constituents (e.g., phase, grain size, morphology) have on hardness, ductility and wear resistance?
- What effects do different physiologically relevant electrolyte compositions (PBS with H_2_O_2_ and HCl additives) have on the electrochemical properties of TiAl6V4?
- Are microstructural constituents of TiAl6V4 prone to dissolution under simulated inflammatory crevice conditions?
- Is cell adhesion affected by the present microstructure of TiAl6V4 alloy?

## 2. Materials and Methods

### 2.1. Sample Preparation and Heat Treatments

TiAl6V4 wrought alloy with extra-low interstitial impurity (TiAl6V4 ELI, ASTM F136 [3], ISO 5832-3 [2]) was purchased as rods at a metal trade (Schnabel GmbH, Braunschweig, Germany). The chemical composition is listed in Table 1 and confirms very low impurities and an accordance with the relevant biomedical material standards. According to the inspection certificate, the material exhibits a 0.2% yield strength of 921 MPa and tensile strength of 989 MPa at an elongation of 19%.

Since Ti-based alloys have a high affinity to O and N above 400 °C, their concentrations before and after the performed heat treatments were measured based on carrier gas melt extraction using the device ON/H-mat 286 (Juwe Laborgeräte GmbH, Viersen, Germany). To exclude surface contaminations, each sample was freshly polished and then placed in a graphite crucible in an inert gas stream and melted in a pulse furnace at a temperature of 2100 °C. The resulting sample gases were transported through the detector system, which consists of a NDIR detector for CO and a thermal conductivity detector for N.

The rods were sectioned by cut-off grinding under liquid cooling lubrication into discs (ø 25 mm × 5 mm, n = 12) and platelets (ø 12 mm × 5 mm, n = 6 for cell tests). Prior to heat treatment, all samples were coated with a ceramic tinder paste (LUISO W61, DAM Härtetechnik GmbH, Ludwigshafen, Germany) for protection against oxidation. 

**Figure 1:**
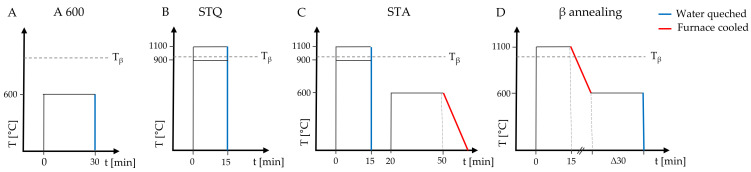
Schematic temperature (T)–time (t) diagrams of the performed heat treatments on the initial TiAl6V4 wrought condition: (**A**) annealing (A 600), (**B**) solution-treated quenched (STQ), (**C**) solution-treated annealed (STA) and (**D**) β annealing. Solution treatment was made above and below the β transus temperature for TiAl6V4 alloy (T_β_, indicated by a dashed grey line).

The temperature–time schematics in Figure 1 show the performed heat treatment processes.

A wrought alloy sample was annealed at 600 °C for 30 min and is referred to as A 600 (Figure 1A). Solution-treated quenched (STQ) samples were placed in a preheated furnace (L9/R, Nabertherm GmbH, Lilienthal, Germany) at 900 or 1100 °C for 15 min and quenched in water (Figure 1B). Since long solution treatment results in excessive grain growth and coarsening of the β phase that negatively affects the mechanical properties, solution treatment was limited to 15 min [24]. The solution treatment temperature was chosen above and below the transus temperature to determine the effect of the remaining primary α phase (<T_β_). Subsequent annealing at 600 °C for 30 min with slow furnace cooling to room temperature resulted in samples referred to as solution-treated annealed (STA, Figure 1C). For β annealing, samples were placed in a furnace at 1100 °C for 15 min, followed by furnace cooling to 600 °C with a cooling rate of 170 °C/h. The annealing temperature of 600 °C was held for 30 min until the sample (β 1100) was water-quenched (Figure 1D).

### 2.2. Metallographic Preparation and Microstructural Investigation

After heat treatments, all samples were metallographically prepared. They were hot-mounted in a conductive phenol resin with carbon additives and manually ground with diamond plates (Plato II, grit 125 µm, Microdiamant AG, Lengwil, Switzerland) under flowing tap water. Afterwards, a lapping step was performed (lapping disc MD-Largo, Struers GmbH, Dresden, Germany) using 9 µm diamond suspension (Meta-Di, Buehler). A scratch- and deformation-free surface was generated by a chemical–mechanical polishing process with a mixture of silicon oxide and hydrogen peroxide (35%) (MasterMet 2, Buehler, Leinfelden-Echterdingen, Germany) on synthetic fiber for 10 min. The roughness of all as-polished samples was measured using a confocal microscope (μsurf expert, NanoFocus AG, Oberhausen, Germany). The polished platelets were manually dismounted from the embedding resin for the cell tests.

For digital optical and scanning electron microscopy (SEM, FEI Scios DualBeam, Thermo Fisher Scientific, Waltham, MA, USA), representative samples were etched in an immersion bath (100 mL of deionized water, 5 mL of hydrogen peroxide and 2 mL of hydrofluoric acid) for 30 s [29]. The SEM imaging contrasts of secondary electrons (SE) for topography and backscattered electrons (BSE) for materials’ composition were applied as well as energy dispersive X-ray spectroscopy (EDS) to determine the chemical composition of microstructural constituents. For detailed structural analysis, electron backscatter diffraction (EBSD) revealed information regarding grain size, crystallographic orientations and phase distribution. 

The obtained electron micrographs in SE and BSE contrast were evaluated using the software ImageJ [30]. Area fractions (indicated as A % in the following) of microstructural constituents (e.g., α and β phase) were determined using a greyscale threshold for phase separation on a total of three images per sample (5000× magnification). The mean grain size of each sample was determined by measuring the grain sizes in digital optical micrographs. Correspondingly, the grain sizes of the two-phase samples were measured in an area of 0.27 × 0.2 mm (50× magnification) and grain sizes of samples solution-treated at 1100 °C were measured in an area of 2.75 × 2.1 mm (5× magnification) of three different locations.

### 2.3. Mechanical Characterization

Mechanical properties were assessed by macro-hardness measurements (HV10) using a nominal test load of 98.07 N (Wilson UH250, Buehler Ltd., Leinfelden-Echterdingen, Germany). Ten individual measurements were performed on each as-polished sample. 

In addition, nanoindentations were performed according to DIN EN ISO 14577-1/-4 (NHT3 nanoindentation tester, Anton Paar TriTec SA, Peseux, Switzerland) [31]. At a constant rate of 20 mN/min, a Berkovich diamond indenter performed quasi-static load-controlled measurements to a maximum of 10 mN. This load was held for 10 s before the relief. Based on the model of Oliver and Pharr [32], indentation modulus, E_IT_, and hardness, H_IT_, were derived from the recorded load-displacement curves. Each sample was indented 10 times with a vertical and horizontal distance between each indent of 100 µm × 100 µm, respectively.

### 2.4. Tribological Tests

The wear resistance of all heat-treated samples was determined in tribological tests against a polished alumina ball (99.9 % Al_2_O_3_, Ø 10 mm). In a ball-on-disc test setup, the alumina ball oscillated against the polished samples’ cross-sections with a normal force of 1 N over a length of 5 mm at 1 Hz under serum lubrication (serum for standardized wear tests with a protein content of 30 g/L according to ISO 14242-1 [33]). For the specified normal force and material combination, the Hertzian contact pressure is about 420 MPa (E_Al2O3_ = 406 GPa, E_TiAl6V4_ = 120 GPa, v_Al2O3_ = 0.23, v_TiAl6V4_ = 0.33). This value exceeds the average contact pressure in taper junctions (40–100 MPa), but is within the range of maximum contact pressure at the taper edges (350–400 MPa) that cause taper wear [34]. Before the test, the arithmetic mean roughness (R_a_) of the final polished sample surfaces was determined by confocal microscopy (R_a_ = 1.8 ± 0.4 nm). The friction coefficient was monitored over a total period of 1000 cycles. Afterwards, samples were cleaned with ethanol in an ultrasonic bath for 10 min and wear volumes of the resulting wear tracks were determined using confocal microscopy (μsurf expert, NanoFocus AG, Oberhausen, Germany).

### 2.5. Corrosion Testing

#### 2.5.1. Electrochemical Tests

The corrosion resistance was investigated by open-circuit potential (OCP) and polarization measurements using a three-electrode arrangement in an electrochemical workstation (Meinsberger Potentiostat PS6, Waldheim, Germany). As the physiological solution, phosphate-buffered saline (PBS, product number: L 1825, Biochrom GmbH, Germany) was used with additives, resulting in a concentration of 30 mM of H_2_O_2_ and 170 mM of HCl (pH = 1), that simulate inflammatory conditions. Before each test, the samples were freshly ground under tap water using a diamond grinding pad (Plato 10 µm, Microdiamant AG, Lengwil, Switzerland) and rinsed with distilled water and ethanol. A chemically resistant film was glued to the surface of the sample, which limited the tested surface to an exposed area of 20 mm^2^. The component system, consisting of the sample, rubber ring and Plexiglas cell, was mechanically sealed by a clamping connection. The sample was contacted, and 3 mL of the above-mentioned electrolyte was filled into the Plexiglas cell. An Ag/AgCl reference electrode and a Pt counter electrode were placed into the cell. OCP was measured at 20 °C for 7200 s. After the stabilization for 7200 s, polarization curves were measured from −200 to 1000 mV_Ag/AgCl_ relative to the value of OCP at a scanning rate of 1 mV/s.

Corrosion potential (E_corr_) and corrosion current density (i_corr_) were calculated by Tafel extrapolation on the cathodic and anodic partial reaction branches (Tafel coefficients β_a_ and β_a_). In addition, the polarization resistance (R_LPR_) was determined by applying the method of linear polarization resistance (LRP) for better comparison. Therefore, the slope of the potential-current density curve over a potential window of ±5 mV around the free corrosion potential (E_corr_) was determined and equivalent to R_LPR_. These values were utilized to determine the corrosion current density, i_LRP_, according to the Stern–Geary relationship [35].

#### 2.5.2. Crevice Corrosion Tests

Six heat-treated sample discs were provided with a centric bore of 7 mm. Crevice corrosion tests were performed according to a previously developed method using PTFE crevice formers [29]. The crevices on the PTFE counterparts exhibited constant widths of 1 mm, crevice lengths varied between 1 and 8 mm and heights ranged from 0.01 to 1 mm. The dimensions represent common crevices of taper junctions of hip endoprostheses based on surface roughness and taper clearance. According to the different crevice sizes, the resulting anode/cathode area ratio varied for the samples between 0.05% (1 mm crevice length) and 0.4% (8 mm crevice length). Inflammatory conditions were simulated by the modified PBS solution with 30 mM of H_2_O_2_ and 170 mM of HCl (pH = 1). The wet assembled samples were stored in an incubator at 37 °C in an aerated condition for four weeks. After immersion testing, samples were dismounted, immediately washed with warm tap water and cleaned in an ultrasonic bath for 10 min using isopropanol. Relevant regions of the surfaces were investigated using SEM.

### 2.6. Cell Adhesion and Spreading

Per sample platelet, 0.3 × 10^5^ SaOs-2 cells (DSMZ, Braunschweig, Germany) were seeded in 100 µL of Dulbecco’s Modified Eagle Medium (DMEM) culture medium (Sigma Aldrich GmbH, Hamburg, Germany) with the addition of 10% fetal calf serum (FCS) and 1% penicillin/streptomycin, and incubated for 2 h at 37 °C. Then, 1 mL of culture medium was added, and the sample was again incubated for 24 h at 37 °C. After the incubation period, the samples were washed two times with PBS, fixed with formalin for 30 min and washed two times again with PBS. Adhering cells were stained with Phalloidin (1:100) for cytoskeleton and DAPI (1:1000) for nucleus for 15 min. Samples were washed two times with PBS and then analyzed using fluorescence microscopy with 40× magnification (Axio Observer Z1, Carl Zeiss GmbH, Jena, Germany), taking five images per sample and experiment. Fluorescence was detected at the wavelength of 455 nm (DAPI blue, Phalloidin yellow/orange). Cells were analyzed with respect to their nucleus (blue fluorescence) and cytoskeleton size (yellow/orange fluorescence) using ImageJ software [30]. Each sample was cleaned using RIPA buffer, trypsin and 70% ethanol, dried and irradiated with UV light overnight. The experiment was repeated three times for each sample.

## 3. Results

### 3.1. Microstructure of Heat-Treated TiAl6V4 Alloy

**Figure 2:**
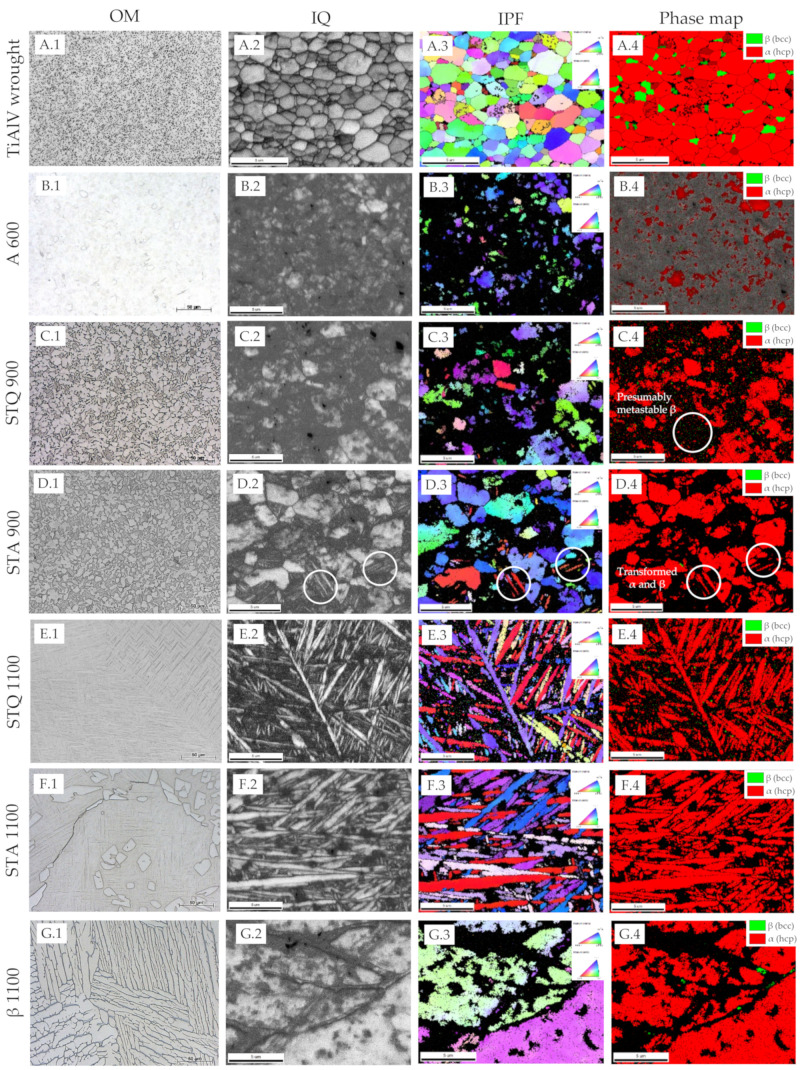
Micrographs and corresponding EBSD results of different conditions of the TiAl6V4 alloy. (**A**) Initial condition of TiAl6V4 wrought alloy and (**B**) aged condition (A 600) at 600 °C for 30 min of former wrought alloy. (**C**) Solution-treated at 900 °C and quenched sample (STQ 900) and (**D**) subsequently aged sample (STA 900). (**E**) Sample after solution treatment at 1100 °C (STQ 1100) and (**F**) afterwards aged (STA 1100). (**G**) β annealed sample previously solution-treated at 1100 °C with partial furnace cooling (ββ 1100). Different representations of the present microstructure are displayed in each column: (**A.1**–**G.1**) digital optical images, measured EBSD data as (**A.2**–**G.2**) image quality map (IQ) illustrates grain boundaries, (**A.3**–**G.3**) inverse pole figure (IPF) map indicates the crystallographic orientation of the grains and (**A.4**–**G.4**) color-coded phase map of the present crystal structure (hcp in red, bcc in green).

**Figure 3:**
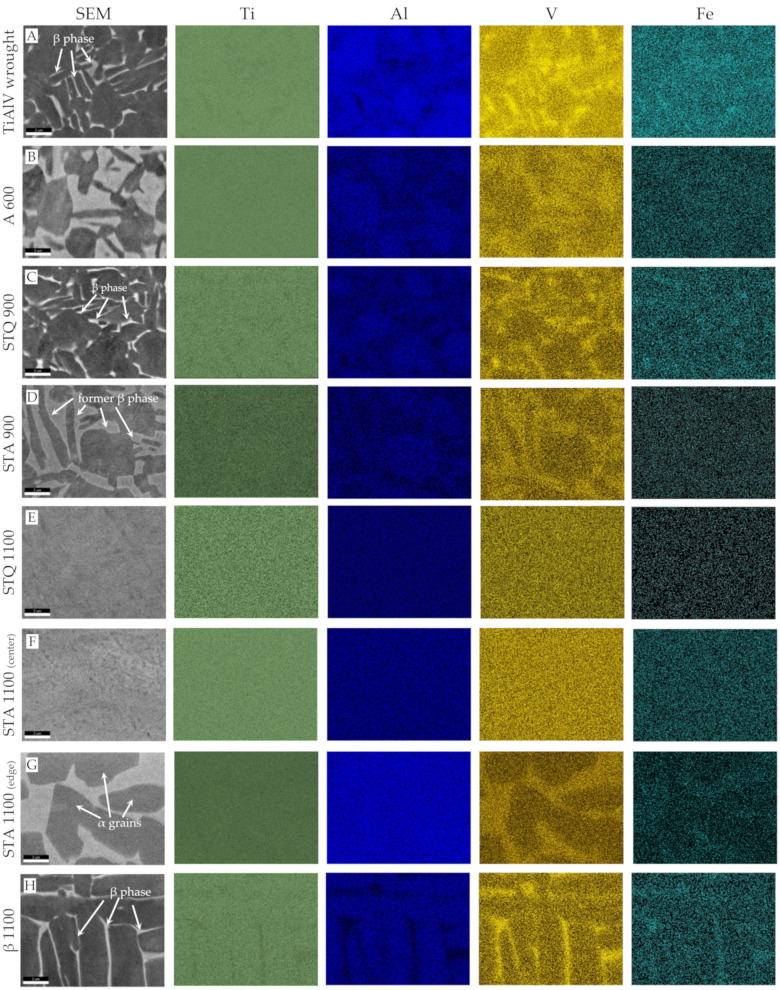
Electron micrographs and EDS maps showing the elemental distribution of Ti, Al, V and Fe in (**A**) the initial condition of TiAl6V4 wrought alloy and after different heat treatments, such as: (**B**) A 600, (**C**) STQ 900, (**D**) STA 900, (**E**) STQ 1100, (**F**,**G**) STA 1100 and (**H**) β 1100.

The microstructure was investigated using optical and electron microscopy combined with EBSD. The obtained micrographs are shown in Figure 2. The initial TiAl6V4 wrought condition exhibits a fine-grained, two-phase microstructure of homogenously distributed β grains of bcc crystal structure embedded in the α matrix of the hcp crystal structure (Figure 2A.1–A.4). The recrystallized α grains have a polyhedral shape with an average dimension of 3.2 ± 2.5 μm and mean size of 12.5 µm^2^. They merge to a larger α phase region, in which sub-micron β grains are located at grain boundaries and triple points, forming thin lamella. The β phase area fraction of the β phase exhibits 14.9 ± 0.2 A% based on graphical analysis. The inverse pole figure (IPF) representation (Figure 2A.3) shows no preferential grain orientation, but a color gradient within single grains demonstrating present plastic deformation in the initial wrought condition. Chemical composition was determined by EDS mapping (Figure 3A). Results of EDS spot analysis demonstrate increased concentrations of Al, as α stabilizer, in α phase, and of V and Fe, as β stabilizers, in β phase (Table 2). Fe impurities originate from the titanium extraction process [23]. The overall content of alloying elements shows a large concentration gradient between the α and β phase, with values of 13.4 m% and 22.2 m%, respectively. Hot gas extraction was used to determine the initial concentration of interstitial N and O and the results verify very low concentrations < 0.02 m% (Table 2).

Annealing at 600 °C of the TiAl6V4 wrought condition (A 600, Figure 2B) leads to a coarsening of the former β grains, as shown by thickened lamellae and enlarged equiaxed β-like grains of 2–4 µm in size, that merge to a homogenous area around primary α grains. An increased mixing of the alloying elements towards the thermodynamically stable equilibrium state of predominant α (hcp) phase occurs between the original phases. Compared to the wrought condition, the concentrations of V and Fe strongly decrease in the β phase to a total alloying content of 15.6 m% (Figure 3B, Table 2). This concentration decrease also destabilizes the prior β phase, which cannot be sufficiently indexed by EBSD due to a strong distortion from the ideal bcc crystal structure (Figure 2F).

After STQ heat treatment, the microstructure changes according to the solution treatment temperature, which is below or above the transition temperature (T_β_). In the case of the samples that were solution-annealed at 900 °C and quenched (STQ 900), electron micrographs demonstrate a characteristic two-phase microstructure similar to the wrought condition (Figure 2C). EBSD results demonstrate that primary α grains remain, as exemplified in Figure 2C.4. It is assumed that metastable ß phase (dark region) began to transform from the grain boundaries, but it cannot be correctly indexed. An uneven grain size distribution is present with α grains in a size range of 1–7 µm and smaller β grains in the form of thin, elongated bars of sub-micron size. The elemental concentration gradient between both phases as well as the interstitial O content are similar to the wrought condition (Table 2).

Samples aged for 30 min at 600 °C after STQ treatment are referred to as STA 900. During aging, the polyhedral primary α grains of STQ 900 remain or slightly decrease in size, as additional α grains are formed, resulting in a mean grain dimension of 5.9 ± 2.3 µm (Figure 2D.4). Areas of metastable β decompose during aging to α and β phase by precipitating fine, incoherent β particles at dislocations or β layers at plate boundaries, as described by Lütjering et al. [23]. STA 900 represents a bimodal microstructure of equiaxed primary α and regions of lamellar α and β matrix. Similar to the annealed wrought condition (A 600), STA 900 treatment leads to grain coarsening, and alloying elements increasingly balance to an equilibrium state. Figure 3D shows enlarged former β grains with a decreased alloying content of 14.7 m% (Al, V and Fe), compared to the quenched STQ 900 sample with 22.3 m% (Table 2).

**Figure A1:**
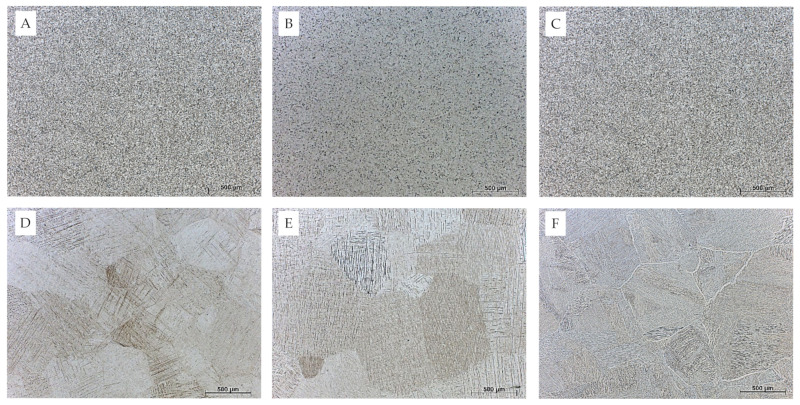
Optical micrographs show the macroscopic microstructures of different TiAl6V4 alloy treatments. Fine-grained conditions are present in the (**A**) wrought alloy, (**B**) A 600 and (**C**) STQ 900 samples, whereas coarse grains with intergranular substructures are present in the samples (**D**) STQ 1100, (**E**) STA 1100 and (**F**) β 1100.

Solution treatment above T_β_ at 1100 °C (STQ 1100) and subsequent quenching leads to the formation of coarse grains with dimensions between 0.5 and 1 mm (Appendix A Figure A1). An acicular martensitic α′ microstructure is present within the grains with minimal fractions of remaining β matrix between martensitic laths (Figure 2E.4). Martensite needles of sub-micron size are intertwined and exhibit recurring orientations visualized by the same coloring in the IPF (Figure 2E.3). Due to almost identical lattice parameters of α Ti and α′ Ti martensite, a corresponding phase differentiation is not possible on the basis of EBSD phase analysis. This fact is particularly evident comparing the phase maps of the samples STQ 900 and STQ 1100 (Figure 2C.4, E.4), in which α and α′ are identically indexed. If the diffraction patterns do not sufficiently match the phases specified by a defined CI threshold, these areas are displayed in grey and black. Very small fractions of ß phase are located in the black-colored regions and between the needles.

**Figure 4:**
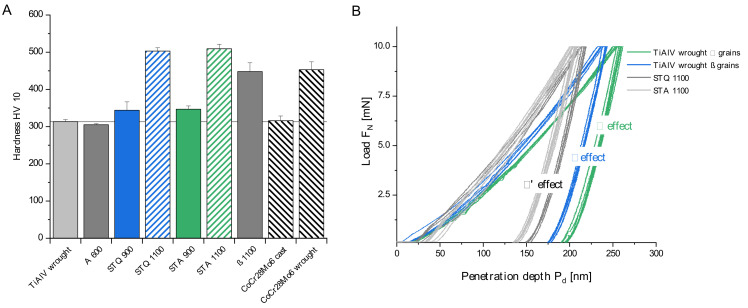
Mechanical properties of heat-treated TiAl6V4 alloy. (**A**) Measured macro-hardness HV10 and (**B**) load-displacement curves from nanoindentation with 10 mN of exemplary TiAl6V4 wrought alloy, STQ 1100 and STA 1100 regarding the effect of present α, α‘ or β phase (data as mean ± SD, n = 10).

After subsequent aging (STA 1100), single α grains (<20 µm in dimension) are predominately embedded along grain boundaries in the acicular microstructure (Figure 2F.1 and Figure 4F,G). An increase in needle thickness to 0.5 to 2 µm can be observed (Figure 2F). A transformation of αȲ martensite into finely divided α and ß grains as a result of aging at 600 °C cannot be clearly observed based on EBSD investigations. Small areas of ß phase are present in STA 1100 and the blackened areas have diminished compared to STQ 1100 (Figure 2E.4,F.4). Increasing temperature during solution treatment raises the fraction of β phase at the expense of α phase fraction. In this β phase, the β stabilizing element V distributes uniformly, causing a V leaner β phase with increasing volume fraction. When the β phase is lean in V (<6 m%) upon rapid cooling, it transforms to martensitic plates that contain a high dislocation density [23]. A complete martensitic microstructure is seen in STQ 1100 and to a dominant fraction in STA 1100 due to a homogenous elemental distribution that destabilizes the β phase (Figure 3E–G, Table 2). Whereas the interstitial N level remains constant < 30 ppm to the initial wrought condition, the interstitial O concentration increases about 4.5 times, to about 130 pm. The high chemical affinity of Ti to O and high solid solubility of O in Ti (about 14.5%) cause the formation of a TiO_2_ layer by oxidation and oxygen diffusion. The oxygen from the environment and the formed oxide layer is dissolved in the bulk material above 550 °C [23]. The results confirm that the high temperature of 1100 °C increases Ti oxidation in contrast to annealing at 600 °C or short-term solution treatment at 900 °C. The absolute values are still below the required oxygen concentration of <0.2 m% according to the standard ISO 5932-3 for surgical TiAl6V4 implants (Table 2) [2].

The ß treatment (β 1100) results in a coarse-grained microstructure with a large amount of lamellae arranged in bundles, also known as basket-weave Widmanstätten microstructure. The grains exhibit a mean size of 0.45 ± 0.36 mm^2^, where the grain boundary is enclosed by an α lamella. The lamellae are separated by stripes, being less than a micron wide, which are assigned to the β phase, as shown in Figure 2G. The IPF representation displays parts of large lamellae with different orientations and the corresponding grain boundaries. During the slow furnace cooling and subsequent annealing process, Al partitions to the α phase, whereas V and Fe segregate to the β phase, causing an increased overall content of alloying elements of 21 ± 1.7 m% in the ß phase. The β phase exhibits an increased V concentration with 14.9 m% and decreased Al with 4.4 m%, compared to the α grains with 3.8 and 7.8 m%, respectively. With sufficiently slow cooling from the β phase field to the α + β field, the α grains initially nucleate along the coarse β grain boundaries and their lamellae grow into the β grains, forming α colonies, as described by Lütjering et al. [23]. Within an α colony, a single α plate is separated by a retained ß matrix, whereby the Burgers relationship, {0001}_α_||{110}_β_ and <1120>_α_||<111>_β_, is obeyed at the flat surface of the forming α discs [23]. Slow furnace cooling is intended to promote growth of α plates that enrich the β phase with β stabilizers. Annealing allows diffusional processes that cause a redistribution of alloying elements, and stabilized β phase is retained at room temperature. The phase-specific composition of the β 1100 specimen corresponds to the two-phase TiAl6V4 wrought condition (Table 2).

### 3.2. Mechanical Characterization

The modified microstructures of TiAl6V4 alloy influence material hardness, as shown in Figure 4A. In its initial wrought condition, the alloy has a hardness of 313 ± 6 HV10. The low standard deviation indicates the presence of a homogenous fine-grained microstructure. Annealing of the wrought condition (A 600) demonstrates a similar macro-hardness of 305 ± 3 HV10. Quenching at a temperature of 900 °C results in a hardness increase of about 10%, to 344 ± 22 HV10. The highest hardness of TiAl6V4 alloy can be achieved by quenching from above T_β_, generating a fine needle-like microstructure. Accordingly, STQ 1100 exhibits a maximum mean hardness of 503 ± 9 HV10, which corresponds to an increase of 61% compared to the wrought condition. The subsequently annealed samples STA 900 and STA 1000 exhibit a similar hardness to the corresponding quenched conditions, with 347 ± 9 and 509 ± 12 HV10, respectively. It can be concluded that the observed grain growth has a negligible effect on hardness and annealing did not cause excessive oxygen absorption (Table 2). An increased hardness of 448 ± 23 HV10 is observed for the partially furnace-cooled β 1100 samples. In addition, CoCr28Mo6 cast and wrought reference samples are displayed and reveal that quenched TiAl6V4 alloy (STQ 1100) can even exceed the high hardness of CoCr28Mo6 wrought alloy.

Nanoindentation can be used to characterize microstructural features and their effect on hardness in the nanoscale. The test load of 10 mN resulted in maximum penetration depths of 180–260 nm, which are appropriate to determine the effect of sub-micron β grains compared to α grains (1–4 µm). The obtained load-displacement curves (Figure 4B) demonstrate clear effects for the present microstructural constituents. As shown in Figure 4B, TiAl6V4 wrought alloy exhibits two distinct curve shapes due to the two phases present. Based on the derived material parameters listed in Table 3, the β phase exhibits a lower mean hardness and indentation modulus of 4.9 and 135.8 GPa compared to the α phase that contributes to increased values of 5.9 and 144.6 GPa, respectively. These results confirm the key differences in crystal structures, according to which the ductile bcc lattice structure of the β phase offers 12 slip systems, as opposed to only 3 for the less ductile, anisotropic character of the hcp α phase [36]. The anisotropy of α grains causes a broader variation of hardness compared to the β phase based on the induced dislocation structure [37]. Liu et al. performed high-speed nanoindentation mappings on near-alpha TA 15 alloy and reported similar phase-specific values that correlate to their elemental distribution. Consequently, significant element partitioning causes hardening due to an increase in average size misfit and solid solution strengthening [38].

The annealed A 600, STA 900 and quenched STQ 900 conditions demonstrate similar two-phase hardness characteristics to the wrought condition. Whereas the indentation moduli of the characteristic phases decrease in A 600, they increase in STA 900. Since both samples exhibit similar phase-specific alloying gradients, this effect can be attributed to the diverging grain sizes and phase morphology. The bimodal microstructure of STA 900 with fractions of primary α grains and fine precipitations of solute lean β phase and α lamellae (aged martensite) have a strengthening effect and increase the elastic moduli, which corresponds to the investigations of Meyer et al. [18]. The difference in elastic modulus between the wrought condition and A 600 reveals its concentration dependency, which is typical for binary Ti-V alloys, as described by Hanada [39] and Fedotov et al. [40].

The samples quenched from above the β transus temperature (STQ 1100 and STA 1100) show reproducible indentation curves (Figure 4B) that correlate with the uniform chemical composition and homogenous acicular microstructure. The samples exhibit an increased modulus with a value range between 149 and 155 GPa and an increased hardness, which can be attributed to higher dislocation density, possible twin formation and the grain refinement by the finely distributed martensitic plates [23,41]. In comparison to similar studies in a vacuum or argon furnace, the increase in interstitial O has a significant effect on hardness, with mean values of about 500 HV10 compared to values of about 400 HV [18,42,43].

For the annealed sample STA 1100, a hardness decrease would be expected compared to the quenched condition based on the observed needle growth in the micron scale. The results verify that the observed microstructural coarsening has a negligible effect on hardness.

Besides the largest grains, the β annealed sample (β 1100) of the Widmanstätten microstructure exhibits a hardness increase compared to the similar TiAl6V4 wrought condition. The hardness increase is due to a significant O uptake during furnace cooling which acts as an α stabilizer and causes significant interstitial strengthening of α phase. This effect compensates α grain coarsening. Similar heat treatments in argon or vacuum atmosphere hinder oxygen diffusion and result in similar hardness and modulus of the two-phase wrought microstructure [18].

### 3.3. Tribological Properties

**Figure 5:**
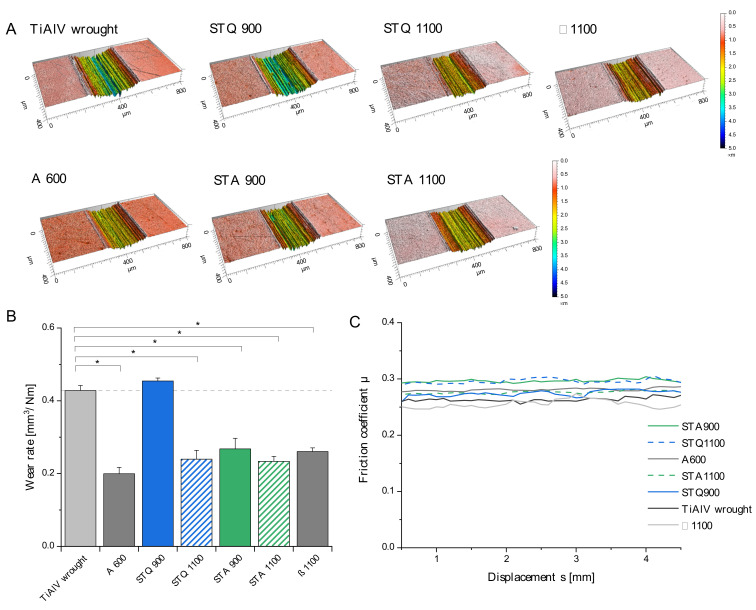
Tribological behavior of the heat-treated TiAl6V4 alloys tested against an alumina ball under serum lubrication. (**A**) Depth-colored confocal images of representative wear tracks generated during the tribological test. (**B**) The calculated mean wear rates show a significant reduction for the heat treated samples A 600, STQ 1100, STA 900, STA 1199 and β 1100 compared to the wrought condition (two-way ANOVA, post-hoc *t*-test,* *p* < 0.05). (**C**) Courses of the monitored mean friction coefficient for the tested samples (data as mean ± SD, n = 3, N = 3).

Wear resistance is a fundamental requirement for implant applications to limit periprosthetic tissue reactions due to the generation of wear particles. Wear tests under simulated physiological conditions were performed using a ball-on-disc setup. For this purpose, the modified TiAl6V4 specimens were tested against an oscillating polished alumina ball under serum lubrication. The resulting wear tracks were analyzed by confocal microscopy and the results are displayed in Figure 5. 

The color-coded wear marks reveal a depth-dependent variation for the tested samples. Accordingly, the TiAl6V4 wrought alloy and STQ 900 samples exhibit the deepest wear profiles. This trend is confirmed by the calculated wear rates (Figure 5B) that demonstrate significantly reduced rates for all heat-treated samples, except STQ 900. The mean friction coefficient varied in a narrow range between 0.25 and 0.31 (Figure 5C), which demonstrates similar friction states under serum lubrication. The abrasive wear rates are not in correlation with the hardness (Figure 4), which highlights that the occurring wear mechanisms are affected by a multitude of intrinsic material properties and systemic factors of the tribo-environment.

Different studies that investigated the tribological performance of modified TiAl6V4 microstructures confirm that the wear quantities can be reduced by heat treatment [42,44,45]. The fine, acicular martensitic microstructure (comparable to STQ 1100) exhibits significantly lower wear rates than TiAl6V4 wrought and cast condition under different tribological test conditions [42,45]. Subsequent annealing in the α + β temperature region demonstrate a similar increase in wear resistance to the presented findings of STA 1100 [46]. It can be attributed to the high hardness (503–509 HV10) of the acicular conditions (STQ 1100 and STA 1100), which provides resistance against abrasion. The associated lower deformation capability of α′ martensite and increased susceptibility to brittle wear did not occur under the selected tribo-test conditions. 

Reda et al. investigated related heat treatments and demonstrated that the Widmanstätten microstructure (comparable to β 1100) exhibits a significantly higher wear resistance than STQ 900 based on a reduced weight loss of about 50% after dry pin-on-ring wear tests [45]. This confirms the present tribological investigations, despite differences in test lubrication and loading. They conclude that the strain levels during the wear process induce a SIM transformation of retained metastable β phase to α′/α″ martensite [47]. This phase transformation dissipates the introduced mechanical energy of the tribo-counterpart with less abrasive damage [45].

The increased α phase fraction of about 85 A% and the small phase dimensions of the wrought and STA 900 condition cause increased ductility, that does not favor the wear resistance measured by the volumetric wear rate. To eliminate the effect of plastic deformation, tribological tests with increased loading should be performed to determine the resulting weight loss.

Comparable tendencies in tribological characteristics can be seen for similar heat treatment conditions, but most studies lack a detailed microstructural analysis and applied different test parameters (e.g., material of counterpart, contact pressure, sliding speed, lubrication), which prevent a direct comparison. In addition, varying wear measurement parameters (e.g., weight loss, wear depth, wear volume) highly affect the obtained results.

### 3.4. Corrosion Behavior

**Figure 6:**
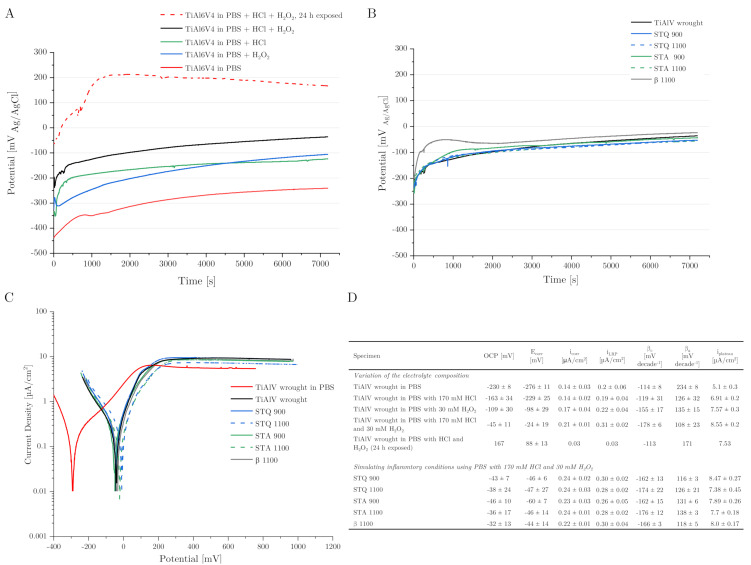
Results of the electrochemical corrosion tests measured against the Ag/AgCl reference electrode. (**A**) Comparison of the electrolyte composition with different additives on OCP of TiAl6V4 wrought alloy. The curves demonstrate the oxidation capacity of HCl and H_2_O_2_ simulating inflammatory conditions. (**B**) OCP measurements of different heat-treated TiAl6V4 conditions in PBS with 170 mM of HCl and 30 mM of H_2_O_2_ immersion for 2 h at room temperature. (**C**) Polarization curves of all tested samples under simulated inflammatory conditions (PBS with 170 mM of HCl and 30 mM of H_2_O_2_). (**D**) Results of the experimental values: OCP (last 300 s), corrosion potential (E_corr_), corrosion current densities according to Tafel extrapolation (i_corr_) or linear polarization resistance (i_LRP_) using the cathodic (β_c_) and anodic (β_a_) Tafel coefficient and high-potential plateau current densities (i_plateau_) of the TiAl6V4 wrought condition in pure PBS, with additives of 170 mM of HCl and 30 mM of H_2_O_2_ and of the heat-treated TiAl6V4 samples under simulated inflammatory conditions using PBS with HCl and H_2_O_2_ (data as mean ± SD, n = 3).

The corrosion behavior of freshly ground TiAl6V4 alloy samples was examined by OCP and polarization measurements against an Ag/AgCl reference electrode. The results of the OCP measurements over an experimental period of 2 h immersion are displayed in Figure 6A,B. Due to the high reproducibility of the results, one representative curve for each sample is shown. The effects of the electrolyte composition on the OCP were measured on the basis of the TiAl6V4 wrought alloy as a reference sample and are illustrated in Figure 6A,D. In PBS, TiAl6V4 wrought alloy exhibits an OCP of −230 mV. Lowering the pH from 7.4 to 1 by adding 170 mM of HCl to PBS resulted in an immediate increase in OCP to −163 mV. A similar oxidation effect occurs by the addition of 30 mM of H_2_O_2_ to PBS, which raises the OCP to −109 mV. The combination of both additives increases the OCP of the wrought condition to −45 mV. A prior 24 h exposure to air-saturated conditions, as is the case with implant components, further increases the OCP to a stagnant value of 167 mV.

The measured OCP for TiAl6V4 wrought alloy corresponds to the typical OCP range of −400 to −200 mV_Ag/AgCl_ measured in PBS, Ringer solution or serum after surface activation [14,28,48]. The increase in OCP of about 200 mV due to combined HCl and H_2_O_2_ addition demonstrates their passivation effect on the sample as well as a stronger cathodic process, which consumes more electrons, resulting in a more positive potential. The contribution of H_2_O_2_ reduction increases the cathodic activity along with an increased surface passivation [13,14]. The results highlight that previous surface treatments, such as exposure to air-saturated conditions, have a significant influence on the stability of the formed oxide layer.

Figure 6B displays that under simulated inflammatory conditions using modified PBS (at a pH of 1 with 30 mM of H_2_O_2_), similar OCP values in the range of −46 to −32 mV occur in all heat-treated TiAl6V4 samples. There is no noticeable difference in mean OCP in comparison to the TiAl6V4 wrought condition used as a reference (Figure 6D). Regardless of the present micro- and macro-structure, the different TiAl6V4 conditions demonstrate similar electrochemical behavior being measured without current against the (Ag/AgCl) reference electrode.

The results of polarization in the anodic direction from −200 to 1000 mV_Ag/AgCl_ relative to the OCP are illustrated in Figure 6C. In PBS or under the simulated inflammatory test conditions, the TiAl6V4 samples do not show a typical active to passive transition, but a plateau region of higher currents than at OCP, which indicates a spontaneous but unstable passive behavior [28]. The respective corrosion current densities (i_corr_/i_LRP_) were derived by Tafel extrapolation on the cathodic and anodic branches (Tafel coefficients β_c_ and β_a_) and are listed in Figure 6D. The cathodic reaction in PBS is oxygen reduction with a low-corrosion current density of 0.14 µA/cm^2^. It can be observed that i_corr_ reduces up to 5 times when the sample is previously exposed to aerated conditions, with the lowest i_corr_ of 0.03 µA/cm^2^. In comparison to the PBS reference, simulated inflammatory conditions (PBS, pH of 1, 30 mM of H_2_O_2_) increase i_corr_ from 0.14 to 0.21 µA/cm^2^ for the wrought condition. The heat-treated TiAl6V4 modifications exhibit similar electrochemical activity, with mean i_corr_ values of 0.22 to 0.24 µA/cm^2^ under inflammatory conditions. These low i_corr_ values indicate a passive state and no active metal dissolution at E_corr_, similar to previous studies [28].

A similar trend can be seen for the corrosion current densities in the plateau region (i_plateau_) at high anodic potentials, which is present between 200 and 1000 mV _Ag/AgCl_ for all samples (Figure 6D). While the lowest i_plateau_ was measured for TiAl6V4 wrought condition in PBS with 5.1 µA/cm^2^, similar to Prestat et al. [14], the inflammatory electrolyte additives HCl and H_2_O_2_ change the shape of the polarization curves and yield higher current densities, with a mean value of 8.55 µA/cm^2^ for their combination. This effect shows an increased dissolution of the Ti-based alloy, due to a less stable corrosion product layer [13,15]. In comparison to the heat-treated samples, the martensitic microstructure of STA 1100 exhibits the lowest i_plateau_, with 7.38 µA/cm^2^.

In summary, the modified TiAl6V4 microstructures show very little differences in corrosion rates using potentiodynamic polarization experiments.

**Figure 7:**
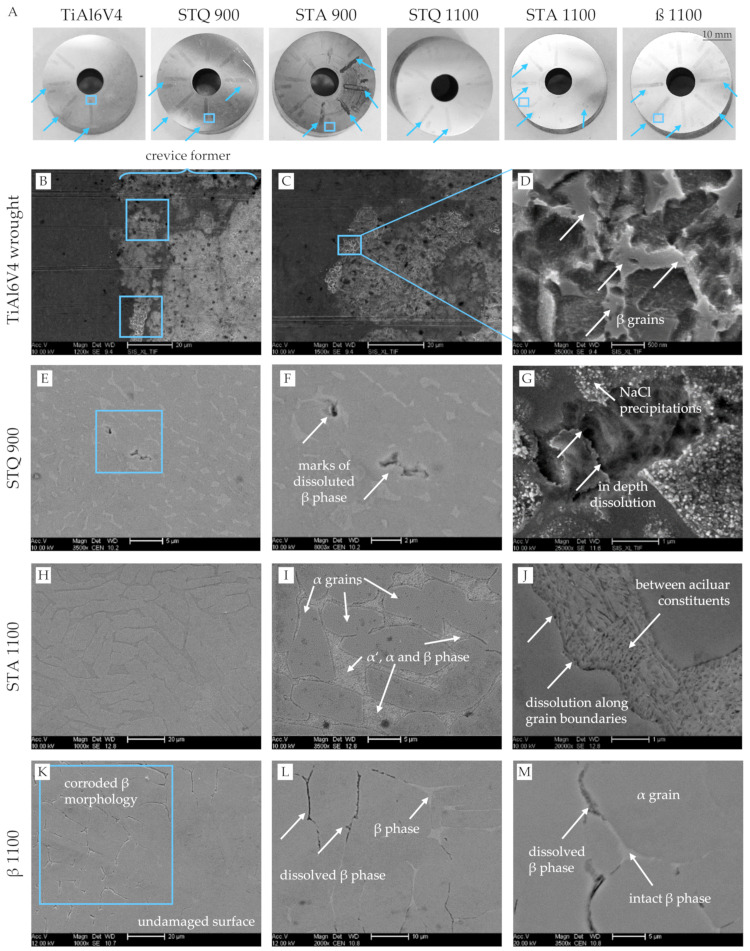
TiAl6V4 sample surfaces after crevice corrosion testing under severe inflammatory conditions simulated by four-week immersion in PBS with HCl and H_2_O_2_ additives at 37 °C. (**A**) Representative photographs show macroscopic crevice-related damaged on heat-treated TiAl6V4 sample surfaces (indicated by blue arrows). All samples are placed with the crevice position 1 at the top [29]. The blue boxes indicate the regions of interests investigated by SEM. (**B**–**M**) Electron micrographs of representative crevice areas for (**B**–**D**) TiAl6V4 wrought alloy indicate a selective α dissolution. In contrast, specific corrosion of β phase and at grain boundaries was observed for the heat-treated samples (**E**–**G**) STA 900, (**H**–**J**) STA 1100 and (**K**–**L**) β 1100.

In addition, the crevice corrosion behavior was tested mimicking inflammatory conditions by four weeks of immersion in PBS with additives, resulting in 170 mM of HCl and 30 mM of H_2_O_2_. Photographs of the resulting sample surfaces are shown in Figure 7A. Crevice-shaped macroscopic discolorations are present on the reference sample as well as on all heat-treated samples, and are most evident for crevice lengths of 8 and 4 mm for crevice heights from 10 to 500 µm. The expression of the markings varies and is very pronounced in the TiAl6V4 wrought alloy, STQ 900 and STA 900 samples. Dark discolorations can be attributed to light interference changes in the corrosion product layer due to H_2_O_2_-containing solution exposure, which was previously reported by other studies on TiAl6V4 alloy [49,50]. Pan et al. studied the electrolyte effect of PBS with H_2_O_2_ addition on cp Ti and described the formation of an oxide bi-layer consisting of a thin, dense layer to the Ti substrate and a thick, porous outer layer [12,51]. In addition, the regions of interest were investigated by SEM and representative micrographs are illustrated in Figure 7B–M.

Micrographs of TiAl6V4 wrought alloy (Figure 7B–D) show corroded areas in the micron scale at the end of the artificially formed crevices by a PTFE former. High-resolution reveals retained bar-shaped β grains and a depletion of equiaxed α grains evidenced by the dark contrast. The results indicate a selective dissolution of the Al-rich α phase for the wrought condition. A recent study by Yu et al. shows a similar localized corrosion of selective α phase dissolution in TiAl6V4 (Grade 5) wrought alloy using 2 M HCl at 37 °C after potentiostatic measurement at −510 mV vs. SCE [52].

Contrary dissolution marks of the β phase were detected in the STQ 900 sample located within the formed crevice front (Figure 7E,F). The BSE contrast clearly demonstrates volumetric dissolution of β grains (light grey) that penetrates in deeper zones along grain boundaries. Figure 7G illustrates an areal surface dissolution next to precipitated sodium chloride (NaCl) crystals resulting from the PBS test fluid.

The quenched, martensitic microstructure (STQ 1100) only reveals macroscopic discolorations (Figure 7A), but no corrosion damage was detected. In comparison to different heat treatments, the homogenous distribution of alloying elements after water quenching seems to exhibit superior crevice corrosion resistance, which was also verified by Geetha et al. for the Ti-13Nb-13Zr system in Ringer solution [53].

The quenched and subsequently annealed sample STA 1100 shows an etched microstructure of globular α grains with remaining fine-needled α′, α and β phase, that is located in the clamping area next to the formed crevice (Figure 7H–J). The micrographs reveal a planar dissolution at the α/β interfaces with local pits between the fine lamellae. Similar results of selective corrosion of β phase were detected in 3.5 M HCl at 37 °C by Atapour et al. [28]. Although a lower concentration of only 170 mM of HCl was used in this study, the limited fluid transport and inhibited re-passivation might have caused an ongoing acidification in the formed crevices resulting in similar selective corrosion degradation.

The β annealed sample (β 1100) exhibits regions at the crevice front and clamp interface that are covered by preferred dissolutions along the β phase (Figure 7K). High-resolution backscattered electron (BSE) micrographs in Figure 7L,M amplify the contrast between α lamellae (dark) and fine β phase (light). They verify a phase-specific dissolution along the β phase, which is illustrated by dark contours next to intact α grains. The typical morphology of long-retained β matrix stripes seems to inhibit an interruption of corrosion procession due to missing α intersections.

Different studies correlate the localized corrosion sensitivity of TiAl6V4 alloy to the preferential dissolution of oxidized V relative to TiO_2_. The generated cation vacancies in the oxide film can diffuse and adsorb ions, such as Cl^−^, which destabilize the corresponding passive layer [54].

Similar localized TiAl6V4 corrosion with β phase depletion under PBS with 100 mM of H_2_O_2_ exposure for five days was documented by Prestat et al. [14]. They observed the formation of a thicker, stable oxide layer on α phase due to oxidation processes and of a porous, defective layer on the β phase, which can be attributed to H_2_O_2_ reduction. It is hypothesized that increased release of V and Fe cations of the β phase enhances the β phase degradation due to Fenton reaction with H_2_O_2_ that generates harmful hydroxyl and hydroperoxyl radicals and due to their contribution to Ti-H_2_O_2_ complexion [14]. Another possible explanation is the formation of micro-galvanic cells within the solute elemental partitioning in the two-phase microstructure. Atapour et al. suggest a preferential corrosion of the β phase based on galvanic corrosion due to the partitioning of V between the α and β phases [28]. Recently, Kurtz et al. presented the concept of cathodically activated corrosion under inflammatory conditions (0.1–3 M H_2_O_2_/PBS solution) causing selective β phase dissolution of TiAl6V4 wrought alloy [55]. They derived a concentration-temperature-time dependency, which corresponds to our observations after four weeks of exposure to critical crevice conditions.

Two effects overlap in the presence of the simulated inflammatory electrolyte. On the one hand, H_2_O_2_ triggers the cathodic activity by its reduction catalyzed by the Ti surface and thickens the present oxide layer, while the corrosion resistance decreases with the increasing H_2_O_2_ concentration [13,14]. On the other hand, strong acidification inhibits the re-passivation of TiAl6V4 alloy, as the oxides dissolve without reforming. Different authors showed a sudden reduction in OCP for acidic electrolytes (e.g., 1.5 M HCl) [28,52,56]. The decrease of the potential is caused by a spontaneous dissolution of the previously formed oxide layer in reducing acidic media [52,57]. The anodic reaction is active Ti dissolution (e.g., Ti^3+^), and water or proton reduction is the likely cathodic reaction in this condition [28]. In this context, hydrolysis properties of the released metal cations (e.g., Ti^2/3+^, V^2/3+^, Fe^2/3+^, Al^3+^) need to be considered as they can reduce water to produce hydrogen ions (H^+^), which further increase the acidity of the solution. In comparison, Fe and V ions exhibit the highest hydrolysis tendencies based on experimental and calculated hydration enthalpies, which measure the cation’s energy to react with water [58].

Due to the combination of H_2_O_2_ and HCl addition to PBS, both effects occur simultaneously. The thin, dense oxide layer of mainly TiO_2_ dissolves progressively, and after weeks of exposure, it is replaced by a more porous and thicker layer of modified Ti oxides with possible incorporation of P-compounds [14]. This remodeling of the passive layer occurs while metal ions are released into the periprosthetic environment. While the main cathodic reaction is oxygen reduction in aqueous solution, the presence of H_2_O_2_ (10–150 mM) changes this pathway [14]. Instead, H_2_O_2_ is reduced, which is even enhanced in acidic medium by the presence of protons. Noguchi et al. show an increased metal ion release in acidic saline solution containing H_2_O_2_ [50]. Another important aspect is the effect of de-aeration, which can occur in the crevices due to stagnant oxygen ingress and can cause a significant potential difference between an aerated (cathodic) and a de-aerated (anodic) sample region. Berbel et al. observed an OCP difference of about 180 mV due to de-aeration in PBS at a pH of 3 and suggested that it might promote galvanic coupling effects [13].

The present results verify that different corrosion processes can occur under simulated inflammatory crevice conditions. On the one hand, micro-crevices inhibit solution transfer and thus cause de-aerated conditions, which further decrease the pH value. The observed selective α phase dissolution resembles the results of different studies that used highly concentrated acid immersion (e.g., 2 M HCl [52]). Based on the findings, it is concluded that selective corrosion of α phase occurs at defined crevice ends (4–8 mm in length × 10–500 µm in height) due to de-aerated conditions that severely increase the H^+^ concentration at the crevice ends compared to the rather low initial HCl content of only 170 mM in PBS. In contrast to other studies, a decrease in OCP using acidified PBS could not be confirmed in the electrochemical measurements (Figure 6A). This shows that the samples remain passive at OCP upon exposure to the very low HCl concentration of 170 mM and no active metal dissolution occurs.

On the other hand, the combination of rather undefined crevice conditions with H_2_O_2_ exposure can also affect the β phase. This is postulated by different factors, such as the release of partitioned Fe, which triggers Fenton reactions in combination with H_2_O_2_ reduction and results in a less protective oxide film compared to α phase, as shown by Prestat et al. [14]. In addition, the β phase exhibits an increased alloying element content and consequently a lower Ti concentration compared to the α phase, which slows the re-passivation tendency and the possible incorporation of P compounds for resealing. The preferential corrosion of microstructural constituents influences the materials’ properties and can possibly harm the mechanical strength due to a notch effect upon implant loading due to tensile stresses, known as stress corrosion cracking (SCC).

### 3.5. Cell Adhesion and Spreading

**Figure 8:**
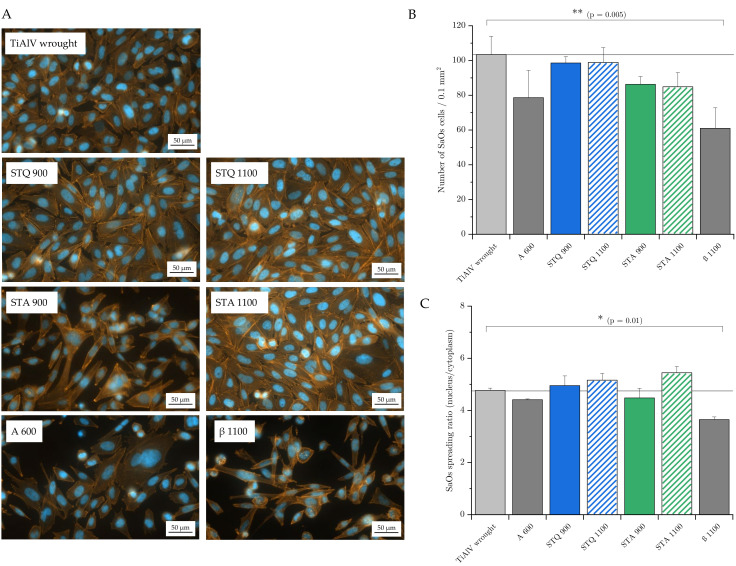
Osteoblast adhesion and spreading to heat-treated TiAl6V4 alloys. SaOs-2 cells were incubated on samples’ surfaces for 24 h at 37 °C. (**A**) Exemplary photographs of SaOs-2 cells adhering to the sample surfaces after incubation (cytoskeleton: orange, nucleus: blue). (**B**) Number of adhering SaOs-2 cells per sample surface of 0.1 mm^2^. (**C**) Size ratio of nucleus to cytoplasm indicating cell spreading for the tested samples (data as mean ± SD, n = 3, N = 5, analyzed for statistical significance with one-way ANOVA with Bonferroni post-hoc test, * *p* < 0.05; ** *p* < 0.01).

Osteoblast-like cells (SaOs-2) were incubated for 24 h at 37 °C on the differently heat-treated samples and on TiAl6V4 wrought alloy to evaluate the effect of microstructural modifications on cell attachment and spreading. The cell attachment was visualized by staining the cytoskeleton with Phalloidin and the nucleus with DAPI. Both substances penetrated the cells and Phalloidin binds selectively to the cytoskeleton’s F-action and DAPI to the minor groove of the double-stranded DNA. Phalloidin is fluorescently labeled with Alexa Fluor 555, which stains the cytoskeleton yellow/orange, and DAPI contains the dye Diamidin-2-phenylindol, which stains the nucleus blue. Representative fluorescent micrographs are depicted in Figure 8A. Similar to the reference TiAl6V4 wrought alloy, the quenched and subsequently annealed samples show spread-out cells adjacent to each other. In contrast, the annealed samples A 600 and β 1100 demonstrate fewer, isolated and smaller osteoblasts. This trend is displayed in the number of adhering SaOs-2 cells in Figure 8B, with a significant decrease in adhering cells for β 1100 compared to the wrought condition (ANOVA with Bonferroni’s post-hoc test, *p* = 0.005). The calculated ratio of nucleus to cytoskeleton size indicates the cell spreading behavior, and mean values are summarized in Figure 8B. The samples that were heat-treated in the β field, STQ 1100 and STA 1100, specify a slightly increased cell-spreading tendency compared to the reference. A significant decrease in the spreading ratio was observed for the annealed β 1100 sample (*p* = 0.01).

Besides the similar integral chemical composition of TiAl6V4 samples, the results reveal a relevant influence of the present microstructure on osteoblast adhesion and spreading. It highlights that the cells seem to be sensitive to the forming passive layer that is determined by the underlying microstructure and exposed medium [12]. The STQ 1100 and STA 1100 samples exhibit a homogenous distribution of alloying elements (Table 2), resulting in a uniform oxide layer which seems to favor cell adhesion and spreading (Figure 8B,C).

The TiAl6V4 wrought, STQ 900 and β annealed (β 1100) conditions exhibit a typical two-phase microstructure with similar β area fraction of 15, 13 and 12 A%, respectively, as well as similar β phase V content of about 15 m% (Table 2). The main differences are the different morphologies of the phase constituents. On the one hand, the wrought and STA 900 conditions exhibit many finely distributed β grains with dimensions < 3 µm. On the other hand, the β 1100 sample has fewer β matrix residues of elongated shape with length scale > 50 µm, exceeding the size of the cell length dimension by about 20 to 50 µm. From the obtained data, it can be assumed that smaller V-rich phase areas are better-tolerated by adhering cells than larger area fractions.

This trend is also confirmed by a decreased cell adhesion behavior on the annealed samples A 600 and STA 900, with a remaining V-rich (~8 m%) phase with area fractions of 40 and 30 A%, respectively. The diffusion processes that occur during annealing at 600 °C destabilize the β phase and favor a homogeneous dissolution of alloying elements in the α phase. Nevertheless, the high V-enriched phase fraction seems to inhibit cell attachment in the present experiment. The minor extent compared to β 1100 can be attributed to the determining role of V as an alloying element and suggests a threshold for enhanced cell–substrate interaction that depends on the V concentration.

This finding corresponds to the study of Challa et al., who showed a decreased cell attachment, proliferation, viability, morphology and spread of pre-osteoblasts on V-containing TiAl6V4 alloy compared to TiAl6Nb7 alloy [59]. Nevertheless, this study missed a characterization of the underlying alloy’s microstructure. The concentration-dependent adverse effect of V compared to Ti and Al particles on cell viability was also demonstrated by Okazaki et al. [60]. To the knowledge of the authors, this is the first detailed study on the microstructural effect of TiAl6V4 alloy on cell attachment and spreading.

## 4. Discussion

This study investigated the microstructure of biomedical-approved TiAlV4 alloy after different heat treatments regarding their mechanical, tribological, corrosion and cell adhesion behavior. The aim was to identify a suitable microstructure that exhibits an increased wear and corrosion resistance while maintaining good cell adhesion.

The results show that the TiAl6V4 microstructure can be widely modified regarding presence, size, morphology and area fraction of phases and microstructural constituents. The microstructure–property relations were examined in detail. It was demonstrated that heat treatments above T_β_ significantly increase hardness in the macro- and micro-scale due to the formation of an acicular martensitic α′ microstructure (STQ 1100, STQ 1100) or an increase in β matrix size (β 1100). Interestingly, the area fraction of β phase remained constant to the wrought condition, which can be attributed to the constant chemical composition. The contribution of O and N uptake on interstitial strengthening due to the high-temperature regime is shown by high values above 500 HV10, in contrast to studies performed in an argon or vacuum atmosphere [18,42,43]. Phase-specific mechanical characteristics were verified by nanoindentations, according to which the α phase has a strengthening effect based on contributions of solid solution and martensite formation, as shown for pure Ti [61].

The tribological behavior was tested simulating implant-related taper micromotion. The obtained wear rates are highest for the wrought and STQ 900 samples, which exhibit similar fine-grained, two-phase microstructures with high volume fractions of primary α phase and ductile behavior. The observed trend revealed that fractions of acicular α or α′ as well as metastable β phase increased the wear resistance, which can be attributed to their higher hardness and decreased ductility. An increase in β phase size as observed for β 1100 causes a lower wear volume. Although wear resistance depends on the system parameters, similar trends of improved tribological behavior due to TiAl6V4 heat treatments were verified in different wear studies [42,45].

**Figure 9:**
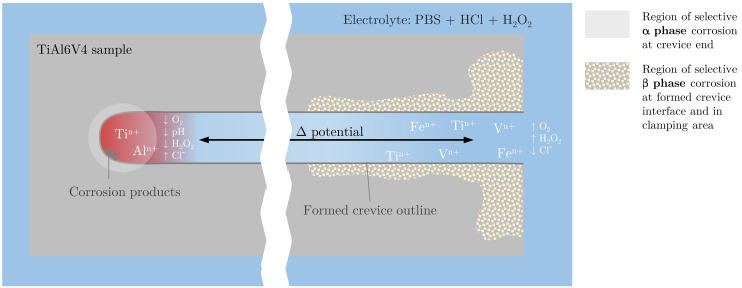
Schematic of the observed corrosion damage locations on TiAl6V4 alloy previously clamped with a PTFE crevice former and immersed for four weeks in PBS with a concentration of 170 mM of HCl and 30 mM of H_2_O_2_ (top view on a TiAl6V4 sample with crevice outline after the disassembly of the PTFE crevice former).

The electrochemical corrosion tests demonstrated that all modified microstructures exhibit similar corrosion rates and characteristics. In contrast, the samples revealed a phase-specific metal dissolution after four weeks of exposure to severe inflammatory crevice conditions. In this scope, different microstructural corrosion processes of TiAl6V4 alloy were duplicated for the first time, which resemble the damage patterns of retrieved modular TiAl6V4 taper junctions [5,6,7]. Critical micrometer-high crevice lengths of 4–8 mm and specific areas that favor selective dissolution of either α or β phase were identified, as illustrated in the schematic in Figure 9. Whereas selective α dissolution occurs at defined crevice ends and is attributed to the instability of Al_2_O_3_ upon excessive acidic conditions due to ongoing deaeration at crevice edges and upon polarization [52,56], the β phase seems to be more sensitive to H_2_O_2_ reduction products, which impairs its re-passivation in areas with undefined crevice geometry due to component clamping [14].

The cell tests revealed a microstructural effect on cell attachment and spreading. Different studies verified the adverse and toxic effect of V oxides on different cells [59,60,62,63,64]. It was hypothesized that a small content of V (3.5 to 4.5 m%) can be tolerated by cells. Areas that exceed this V content threshold inhibit cell adhesion.

This study showed that the biomedical-approved composition of TiAl6V4 alloy can significantly hinder cell adhesion and consequent growth. Based on the results, an appropriate processing of TiAl6V4 alloy is of the utmost importance for implant application. The most relevant finding is the phase-specific dissolution of either α or β phase of TiAl6V4 alloy under inflammatory crevice conditions, which typically occur in modular endoprostheses.

Since the metal ion release caused by corrosion processes impairs the implant service life and can cause inflammatory reactions, further research is required to examine the detailed corrosion damage mechanisms. For this reason, the effect of varying electrolyte composition (e.g., HCl content, proteins) and crevice conditions (e.g., deaeration) must be systematically studied. In addition, advanced in situ methods (e.g., TEM, XPS, EIS) can clarify the processes of selective α or ß dissolution considering the effects of internal residual stresses from mechanical processing, galvanic contribution due to V partitioning, as well as of impurities (e.g., N and C) on oxide layer stability. In this scope, sensorization of the crevice test setup is planned as well as to specifically simulate critical crevice states via polarization conditions to obtain more information on the ongoing selective corrosion mechanisms. Kinetic simulation of the influencing parameters, including crevice geometry, polarization effects, time and species enrichments (e.g., pH change, oxygen depletion), can model the observed selective corrosion processes and quantify their specific impacts. Future studies are planned to investigate the detailed wear mechanisms under varying tribological parameters (e.g., contact pressure, sliding speed, lubricant). The results show that heat treatments predominately modify the phase morphology, but not the phase content of TiAl6V4 alloy. To increase the volume fraction of the β phase in Ti-based alloys, the chemical composition should be adjusted by alloying with β stabilizing elements, such as Mo, Nb, Ta and Cr.

The developed crevice corrosion test method should be preferred in contrast to the current standard ASTM F2129 [65] for electrochemical implant testing using the short-term potentiodynamic polarization test in PBS, which is not representative for severe electrochemical crevice conditions. Implant materials have to withstand critical environments in vivo, such as chronic inflammation resulting in an acidic pH shift and production of ROS [10], which cannot be sufficiently simulated in electrochemical corrosion tests. The periprosthetic environment should be better-mimicked by an adapted electrolyte, critical crevice geometries and sufficient exposure time to identify realistic data on the corrosion degree that can occur in vivo. The presented crevice test method and solution reconstructs the severity of in vivo corrosion. It should be used to evaluate the suitability and safety of metallic biomaterials and implant designs.

The obtained results highlight the enormous potential of a homogenous acicular martensitic α′ microstructure of the frequently used TiAl6V4 implant alloy to avoid selective corrosion in the taper junction of modular endoprostheses. In the context of regular allowance, this thermal treatment can be applied on taper surfaces to increase the wear and corrosion resistance. This material-related optimization seems promising for modular arthroplasty components and can contribute to reduce the risk of premature implant failure.

## 5. Conclusions

Different thermal treatments of TiAl6V4 alloy for orthopedic implant application were investigated and new perspectives of the occurring phase-specific in vivo degradation were achieved under simulated inflammatory crevice conditions. The findings demonstrated that hardness, corrosion susceptibility, wear resistance and cell attachment were improved by homogenous acicular martensitic α′ microstructures as a result of quenching from the ß field region. Manufacturers should pay special attention to the processing, as elemental partitioning between the α and β phase can cause adverse effects regarding an inhibited cell attachment and increased phase-selective ion release under severe inflammatory crevice conditions.

## Figures and Tables

**Table 1:** Chemical composition of the TiAl6V4 ELI wrought alloy according to the corresponding standard and based on the trader’s inspection certificate (m%).

Implant Material	Standard	Ti	Al	V	Fe	O	N	C	H
TiAl6V4 wrought alloy, ELI	ISO 5832-3, ASTM F136	Balance	5.5–6.5	3.5–4.5	≤0.25	≤0.13	≤0.05	≤0.08	≤0.0125
TiAl6V4 ELI (certificate)		Balance	6.06	4.14	0.2	0.11	0.01	<0.01	0.01

**Table 2:** Grain size (µm^2^), area fraction (A %) and chemical composition (m %) of present microstructural constituents of different heat-treated TiAl6V4 samples determined for Ti, Al, V and Fe by EDS spot analysis. Integral value is measured over a sample area of 2 × 2 mm and integral N and O content is determined by gas melt extraction analysis (data as mean ± SD, n = 5 for EDS, n = 3 for gas analysis). A comparison with the chemical composition according to the ISO 5832-3 standard for wrought alloy TiAl6V4 surgical implants is provided [2].

Sample	Grain Size (µm^2^)	Area Fraction (A %)	Ti (m %)	Al (m %)	V (m %)	Fe (m %)	Total Alloying Elements (m%)	N (m%)	O (m%)
ISO 5832-3 *			balance	5.5–6.75	3.5–4.5	≤0.3	9–11.6	<0.05	<0.2
TiAl6V4 wrought (integral) untreated control condition			88.9 ± 0.4	7.0 ± 0.1	3.8 ± 0.3	0.3 ± 0.1	11.1 ± 0.4	0.002 ± 0.001	0.003 ± 0.001
α phase	12.5 ± 6.2	85.1 ± 0.8	86.5 ± 0.2	8.0 ± 0.1	4.7 ± 0.2	0.7 ± 0.2	13.4 ± 0.5		
β phase	0.9 ± 0.3	14.9 ± 0.2	77.7 ± 1.6	4.7 ± 0.2	15.7 ± 1.5	1.8 ± 0.1	22.2 ± 1.8		
A 600 (integral)	42 ± 21		88.8 ± 0.4	7.0 ± 0.1	3.8 ± 0.1	0.3 ± 0.1	11.1 ± 0.3	0.004 ± 0.002	0.003 ± 0.001
Primary α phase		57.8 ± 1.3	86.6 ± 0.5	7.7 ± 0.1	4.7 ± 0.2	0.7 ± 0.1	13.1 ± 0.3		
β-like phase		42.2 ± 1.4	84.3 ± 0.1	6.1 ± 0.1	8.5 ± 0.1	1.0 ± 0.1	15.6 ± 0.3		
STQ 900 (integral)	58 ± 21		88.4 ± 0.3	7.0 ± 0.1	4.2 ± 0.3	0.3 ± 0.1	11.5 ± 0.5	0.005 ± 0.001	0.003 ± 0.001
Primary α phase		87.3 ± 0.5	86.5 ± 0.2	8.0 ± 0.1	4.7 ± 0.2	0.4 ± 0.2	13.1 ± 0.5		
α, α′ and β phase		12.7 ± 0.3	77.7 ± 1.6	4.7 ± 0.2	15.7 ± 1.6	1.9 ± 0.2	22.3 ± 2		
STA 900 (integral)	35 ± 28		89.3 ± 0.3	6.6 ± 0.1	3.8 ± 0.1	0.3 ± 0.1	10.7 ± 0.3	0.005 ± 0.001	0.003 ± 0.001
Primary α phase		70.1 ± 1.1	87.8 ± 0.1	7.3 ± 0.2	4.2 ± 0.2	0.6 ± 0.1	12.1 ± 0.4		
α, α′ and β phase		29.9 ± 0.6	85.3 ± 0.2	5.8 ± 0.1	8.0 ± 0.1	0.9 ± 0.1	14.7 ± 0.3		
STQ 1100 (integral)	245,000 ± 160,000		89.8 ± 0.3	6.8 ± 0.2	3.4 ± 0.3	0.3 ± 0.1	10.5 ± 0.6	0.002 ± 0.001	0.013 ± 0.002
STA 1100 (integral)	475,000 ± 350,000		89.3 ± 0.1	6.8 ± 0.1	3.6 ± 0.1	0.3 ± 0.1	10.7 ± 0.3	0.002 ± 0.001	0.013 ± 0.003
Single α grains			88.4 ± 0.2	7.7 ± 0.1	3.2 ± 0.1	0.6 ± 0.1	11.5 ± 0.3		
ß 1100 (integral)	454,000 ± 357,500		89.3 ± 0.2	6.9 ± 0.1	3.7 ± 0.2	0.3 ± 0.1	10.9 ± 0.4	0.005 ± 0.003	0.013 ± 0.002
α phase		88.2 ± 0.8	87.7 ± 0.1	7.8 ± 0.1	3.8 ± 0.2	0.6 ± 0.1	12.2 ± 0.4		
β phase		11.8 ± 0.3	79.1 ± 0.9	4.4 ± 0.4	14.9 ± 1.1	1.7 ± 0.2	21.0 ± 1.7		

* In addition, the hydrogen content is limited to max. 0.015 m% and the carbon content is restricted to max. 0.08 m%.

**Table 3:** Hardness, H_IT_, and indentation modulus, E_IT_, derived from nanoindentations show a distinct effect for the phases present in the heat-treated TiAl6V4 alloys (data as mean ± SD, n = 10). All samples were heat-treated in air and only one reference sample, β 1100, was heat-treated in argon atmosphere.

Sample with Phase Effects	H_IT_ (GPa)	E_IT_ (GPa)
TiAl6V4 wrought		
α phase	5.9 ± 0.6	144.6 ± 6.9
β phase	4.9 ± 0.1	135.8 ± 5.7
A 600		
primary α phase	6.1 ± 0.1	141.6 ± 1.2
β-like phase	4.9 ± 0.1	133.1 ± 6.91
STQ 900		
primary α phase	6.1 ± 0.4	143.0 ± 8.2
β phase	5.5 ± 0.2	136.6 ± 7.1
STA 900		
primary α phase	6.0 ± 0.3	147.1 ± 6.5
α, α′ and β phase	5.0 ± 0.1	138.1 ± 6.1
STQ 1100 (martensite)	6.6 ± 0.4	164.8 ± 9.9
STA 1100 (martensite)	7.3 ± 0.5	148.8 ± 9.8
β 1100 (air)		
α phase	8.5 ± 0.5	161.3 ± 7.2
β phase	6.2 ± 0.3	135.9 ± 7.1
β 1100 (argon atmosphere)		
α phase	5.8 ± 0.1	149.7 ± 6.5
β phase	4.6 ± 0.2	140.8 ± 2.7

## Data Availability

The data that support the findings of this study are available from the corresponding author upon reasonable request.

## References

[1] Grimberg A., Jansson V., Lützner J., Melsheimer O., Morlock M., Steinbrück A. (2021). EPRD Jahresbericht 2021.

[2] (2017). Implants for Surgery Metallic Materials. Part 3: Wrought Titanium 6-Aluminium 4-Vanadium Alloy.

[3] ASTM International, F04 Committee (2021). Specification for Wrought Titanium-6Aluminum-4Vanadium ELI (Extra Low Interstitial) Alloy for Surgical Implant Applications (UNS R56401): ASTM F136-13(2021)e1(F136).

[4] ASTM International, F04 Committee (2020). ASTM F1472-20a: Specification for Wrought Titanium-6Aluminum-4Vanadium Alloy for Surgical Implant Applications (UNS R56400)(F1472).

[5] Rodrigues D.C., Urban R.M., Jacobs J.J., Gilbert J.L. (2009). In vivo severe corrosion and hydrogen embrittlement of retrieved modular body titanium alloy hip-implants. J. Biomed. Mater. Res..

[6] Gilbert J.L., Mali S., Urban R.M., Silverton C.D., Jacobs J.J. (2012). In vivo oxide-induced stress corrosion cracking of Ti-6Al-4V in a neck-stem modular taper: Emergent behavior in a new mechanism of in vivo corrosion. J. Biomed. Mater. Res..

[7] Hall D.J., Pourzal R., Della Valle C.J., Galante J.O., Jacobs J.J., Urban R.M., Greenwald A.S., Kurtz S.M., Lemons J.E., Mihalko W.M. (2015). Corrosion of Modular Junctions in Femoral and Acetabular Components for Hip Arthroplasty and Its Local and Systemic Effects. Modularity and Tapers in Total Joint Replacement Devices.

[8] Luqman M., Seikh A.H., Sarkar A., Ragab S.A., Mohammed J.A., Ijaz M.F., Abdo H.S. (2020). A Comparative Study of the Electrochemical Behavior of α and β Phase Ti6Al4V Alloy in Ringer’s Solution. Crystals.

[9] Prestat M., Thierry D. (2021). Corrosion of titanium under simulated inflammation conditions: Clinical context and in vitro investigations. Acta Biomater..

[10] Valko M., Morris H., Cronin M.T.D. (2005). Metals, toxicity and oxidative stress. Curr. Med. Chem..

[11] Mu Y., Kobayashi T., Sumita M., Yamamoto A., Hanawa T. (2000). Metal ion release from titanium with active oxygen species generated by rat macrophagesin vitro. J. Biomed. Mater. Res..

[12] Pan J., Liao H., Leygraf C., Thierry D., Li J. (1998). Variation of oxide films on titanium induced by osteoblast-like cell culture and the influence of an H2O2 pretreatment. J. Biomed. Mater. Res..

[13] Berbel L.O., Banczek E.d.P., Karoussis I.K., Kotsakis G.A., Costa I. (2019). Determinants of corrosion resistance of Ti-6Al-4V alloy dental implants in an In Vitro model of peri-implant inflammation. PLoS ONE.

[14] Prestat M., Vucko F., Holzer L., Thierry D. (2021). Microstructural aspects of Ti6Al4V degradation in H2O2-containing phosphate buffered saline. Corros. Sci..

[15] Yu F., Addison O., Davenport A.J. (2015). A synergistic effect of albumin and H₂O₂ accelerates corrosion of Ti6Al4V. Acta Biomater..

[16] Leyens C., Peters M. (2003). Titanium and Titanium Alloys: Fundamentals and Applications.

[17] El-Hadad S., Nady M., Khalifa W., Shash A. (2018). Influence of heat treatment conditions on the mechanical properties of Ti–6Al–4V alloy. Can. Metall. Q..

[18] Meyer L.W., Krüger L., Sommer K., Halle T., Hockauf M. (2008). Dynamic strength and failure behavior of titanium alloy Ti-6Al-4V for a variation of heat treatments. Mech. Time Depend. Mater..

[19] Oh S.-T., Woo K.-D., Kim J.-H., Kwak S.-M. (2017). The Effect of Al and V on Microstructure and Transformation of β Phase during Solution Treatments of Cast Ti-6Al-4V Alloy. Korean J. Met. Mater..

[20] Wanying L., Yuanhua L., Yuhai C., Taihe S., Singh A. (2017). Effect of Different Heat Treatments on Microstructure and Mechanical Properties of Ti6Al4V Titanium Alloy. Rare Met. Mater. Eng..

[21] Oliveira N.T.C., Guastaldi A.C. (2009). Electrochemical stability and corrosion resistance of Ti-Mo alloys for biomedical applications. Acta Biomater..

[22] Oliveira N.T., Aleixo G., Caram R., Guastaldi A.C. (2007). Development of Ti–Mo alloys for biomedical applications: Microstructure and electrochemical characterization. Mater. Sci. Eng. A.

[23] Lütjering G., Williams J.C. (2007). Titanium: With 51 Tables.

[24] Kolli R., Devaraj A. (2018). A Review of Metastable Beta Titanium Alloys. Metals.

[25] Nikolova M.P., Yankov E.H., Da Silva L.F.M. (2019). Corrosion Study of Ti5Al4V and Ti6Al4V in Different Simulated Body Fluids. Materials Design and Applications II.

[26] Dull D.L., Raymond L. (1973). Thermal and Mechanical Effects on the Corrosion Behavior of Ti-6Al-4V Alloy. J. Electrochem. Soc..

[27] Ryba J., Kawalec M., Tyrala E., Krawiec H. (2019). Effect of Thermal Treatment of Biomedical Ti-6Al-4V Alloy on Corrosion Resistance in Simulated Physiological Solutions. Arch. Metall. Mater..

[28] Atapour M., Pilchak A., Frankel G.S., Williams J.C., Fathi M.H., Shamanian M. (2010). Corrosion Behavior of Ti-6Al-4V with Different Thermomechanical Treatments and Microstructures. CORROSION.

[29] Herbster M., Rosemann P., Michael O., Harnisch K., Ecke M., Heyn A., Lohmann C.H., Bertrand J., Halle T. (2022). Microstructure-dependent crevice corrosion damage of implant materials CoCr28Mo6 TiAl6V4 and REX 734 under severe inflammatory conditions. J. Biomed. Mater. Res..

[30] Rueden C.T., Schindelin J., Hiner M.C., DeZonia B.E., Walter A.E., Arena E.T., Eliceiri K.W. (2017). ImageJ2: ImageJ for the next generation of scientific image data. BMC Bioinform..

[31] (2015). Metallic Materials—Instrumented Indentation Test for Hardness and Materials Parameters—Part 1: Test Method (ISO 14577-1:2015); German Version EN ISO 14577-1:2015 (DIN EN ISO 14577-1:2015-11).

[32] Oliver W.C., Pharr G.M. (1992). An Improved Technique for Determining Hardness and Elastic Modulus Using Load and Displacement Sensing Indentation Experiments.

[33] (2016). Implants for Surgery—Wear of Total Hip-Joint Prostheses (14242-1).

[34] English R., Ashkanfar A., Rothwell G. (2016). The effect of different assembly loads on taper junction fretting wear in total hip replacements. Tribol. Int..

[35] Stern M., Geaby A.L. (1957). Electrochemical Polarization. J. Electrochem. Soc..

[36] Donachie M.J. (2000). Titanium: A Technical Guide.

[37] Viswanathan G.B., Lee E., Maher D.M., Banerjee S., Fraser H.L. (2005). Direct observations and analyses of dislocation substructures in the α phase of an α/β Ti-alloy formed by nanoindentation. Acta Mater..

[38] Liu Z., Zhang J., He B., Zou Y. (2021). High-speed nanoindentation mapping of a near-alpha titanium alloy made by additive manufacturing. J. Mater. Res..

[39] Hanada S., Ozaki T., Takahashi E., Watanabe S., Yoshimi K., Abumiya T. (2003). Composition Dependence of Young’s Modulus in Beta Titanium Binary Alloys. Mater. Sci. Forum..

[40] Fedotov S.G., Jaffee R.I., Burte H.M. (1973). Peculiarities of Changes in Elastic Properties of Titanium Martensite. Titanium Science and Technology.

[41] Cai J., Li F., Liu T., Chen B. (2011). Investigation of mechanical behavior of quenched Ti–6Al–4V alloy by microindentation. Mater. Charact..

[42] Cvijovic-Alagic I., Mitrovic S., Cvijovic Z., Veljovic D., Babic M., Rakin M. (2009). Influence of the Heat Treatment on the Tribological Characteristics of the Ti-based Alloy for Biomedical Applications. Tribol. Ind..

[43] Fan Y., Tian W., Guo Y., Sun Z., Xu J. (2016). Relationships among the Microstructure, Mechanical Properties, and Fatigue Behavior in Thin Ti6Al4V. Adv. Mater. Sci. Eng..

[44] Youssef S.S.S., Ibrahim K.M., Abdel-Karim M. (2013). Effect of heat treatment process on tribological behavior of Ti-6Al-4V Alloy. Int. J. Mech. Eng. Robot. Res..

[45] Reda R., Nofal A., Hussein A.-H., El-Banna E.-S. (2014). Tailoring of microstructure of Ti-6Al-4V implant castings for abrasive wear resistance. Int. J. Metall. Mater. Sci. Eng..

[46] Ganesh B.K.C., Ramanaih N., Chandrasekhar Rao P.V. (2012). Dry Sliding Wear Behavior of Ti–6Al–4V Implant Alloy Subjected to Various Surface Treatments. Trans. Indian Inst. Met..

[47] Ganesh B.K.C. (2012). Effect of heat treatment on dry sliding wear of titanium-aluminum- vanadium (Ti-6Al-4V) implant alloy. J. Mech. Eng. Res..

[48] Carsten K. (2018). Einfluss der Wärmebehandlung auf Gefüge, Korrosionsbeständigkeit und Biokompatiblität von CoCrMo-Legierungen. Master’s Thesis.

[49] Noguchi T., Takemoto S., Hattori M., Yoshinari M., Kawada E., Oda Y. (2008). Discoloration and dissolution of titanium and titanium alloys with immersion in peroxide- or fluoride-containing solutions. Dent. Mater. J..

[50] Takemoto S., Hattori M., Yoshinari M., Kawada E., Oda Y. (2013). Discoloration of titanium alloy in acidic saline solutions with peroxide. Dent. Mater. J..

[51] Pan J., Thierry D., Leygraf C. (1994). Electrochemical and XPS studies of titanium for biomaterial applications with respect to the effect of hydrogen peroxide. J. Biomed. Mater. Res..

[52] Yu F., Addison O., Davenport A. (2022). Temperature-Dependence Corrosion Behavior of Ti6Al4V in the Presence of HCl. Front. Mater..

[53] Geetha M., Kamachi Mudali U., Gogia A., Asokamani R., Raj B. (2004). Influence of microstructure and alloying elements on corrosion behavior of Ti–13Nb–13Zr alloy. Corros. Sci..

[54] Metikoš-Huković M., Kwokal A., Piljac J. (2003). The influence of niobium and vanadium on passivity of titanium-based implants in physiological solution. Biomaterials.

[55] Kurtz M.A., Khullar P., Gilbert J.L. (2022). Cathodic Activation and Inflammatory Species Are Critical to Simulating In Vivo Ti-6Al-4V Selective Dissolution. Acta Biomater..

[56] Ruzickova M., Hildebrand H., Virtanen S. (2005). On the Stability of Passivity of Ti-Al Alloys in Acidic Environment. Z. Für Phys. Chem..

[57] Blackwood D.J., Peter L.M., Williams D.E. (1988). Stability and open circuit breakdown of the passive oxide film on titanium. Electrochim. Acta.

[58] Smith D.W. (1977). Ionic hydration enthalpies. J. Chem. Educ..

[59] Challa V.S.A., Mali S., Misra R.D.K. (2013). Reduced toxicity and superior cellular response of preosteoblasts to Ti-6Al-7Nb alloy and comparison with Ti-6Al-4V. J. Biomed. Mater. Res. A.

[60] Okazaki Y., Rao S., Asao S., Tateishi T., Katsuda S., Furuki Y. (1998). Effects of Ti, Al and V Concentrations on Cell Viability. Mater. Trans. JIM.

[61] Kao Y.L., Tu G.C., Huang C.A., Liu T.T. (2005). A study on the hardness variation of α- and β-pure titanium with different grain sizes. Mater. Sci. Eng. A.

[62] Okazaki Y., Rao S., Ito Y., Tateishi T. (1998). Corrosion resistance, mechanical properties, corrosion fatigue strength and cytocompatibility of new Ti alloys without Al and V. Biomaterials.

[63] Molinuevo M.S., Cortizo A.M., Etcheverry S.B. (2008). Vanadium (IV) complexes inhibit adhesion, migration and colony formation of UMR106 osteosarcoma cells. Cancer Chemother Pharm..

[64] Hosseini M.-J., Seyedrazi N., Shahraki J., Pourahmad J. (2012). Vanadium induces liver toxicity through reductive activation by glutathione and mitochondrial dysfunction. Adv. Biosci. Biotechnol..

[65] ASTM International, F04 Committee (2019). ASTM FF2129-19a Test Method for Conducting Cyclic Potentiodynamic Polarization Measurements to Determine the Corrosion Susceptibility of Small Implant Devices.

